# Pectin-Lyase-Modified Ginseng Extract and Ginsenoside Rd Inhibits High Glucose-Induced ROS Production in Mesangial Cells and Prevents Renal Dysfunction in db/db Mice

**DOI:** 10.3390/molecules26020367

**Published:** 2021-01-12

**Authors:** Eunsoo Jung, Mi-kyung Pyo, Junghyun Kim

**Affiliations:** 1 Laboratory of Toxicology, Research Institute for Veterinary Science and College of Veterinary Medicine, Seoul National University, Seoul 08826, Korea; ozz79@snu.ac.kr; 2 International Ginseng and Herb Research Institute, 25 Insamgwangjang-ro, Geumsan-eup, Geumsan-gun 32724, Chungcheongnam-do, Korea; mkpyginherb@gmail.com; 3 Department of Oral Pathology, School of Dentistry, Jeonbuk National University, Jeonju 54896, Korea; 4 Korea Institute of Oriental Medicine, Daejeon 34054, Korea

**Keywords:** diabetes, diabetic nephropathy, ginseng, GS-E3D

## Abstract

Diabetes increases the incidence rate of chronic renal disease. Pectin-lyase-modified ginseng (GS-E3D), with enhanced ginsenoside Rd content, has been newly developed. In this study, renal protective roles of GS-E3D in type-2 diabetic db/db mice were investigated. The generation of reactive oxygen species (ROS) induced by high glucose (25 mM) was reduced by ES-E3D (75%) and ginsenoside Rd (60%). Diabetic db/db mice received 100 or 250 mg/kg/day of GS-E3D daily via oral gavage for 6 weeks. Albuminuria and urinary 8-hydroxy-2′-deoxyguanosine (8-OhdG, an oxidative stress marker) levels were increased in db/db mice and the levels recovered after GS-E3D treatment. In renal tissues, TUNEL-positive cells were decreased after GS-E3D treatment, and the increased apoptosis-related protein expressions were restored after GS-E3D treatment. Therefore, GS-E3D has a potent protective role in diabetes-induced renal dysfunction through antioxidative and antiapoptotic activities. These results may help patients to select a dietary supplement for diabetes when experiencing renal dysfunction.

## 1. Introduction

Patients with diabetes experience hyperglycemia; further exacerbation can result in renal dysfunction and diabetic nephropathy [1]. Multiple epidemiologic studies have shown that diabetic nephropathy is frequent in patients with diabetes, presenting significantly reduced renal function [2]. Consequentially, this worsens their quality of life [3]. There are reports that patients with diabetes present an increased risk of nephropathy, estimated at 2- to 3-fold [4]. Nephropathy with diabetes could be a reason for the development of other severe complications, including proteinuria, glomerulosclerosis, and chronic renal diseases [5]. Therefore, it is important not only to control blood glucose but to have combinative treatment for nephropathy as well.

The causes of diabetic nephropathy are not yet fully understood. However, diabetes is associated with macro- and microvascular abnormalities. Therefore, deterioration of microcirculation and disturbances in glycemic control may lead to degenerative changes in renal tissues [6]. In addition, there are pieces of literature demonstrating that rats with diabetes induced by streptozotocin show increased oxidative stress in their kidneys [7,8,9]. Several strategies to ameliorate ROS production using antioxidants have already been investigated in various second complications of diabetes, including mitochondrial oxidative stress [10], peripheral nerve dysfunction [11], nephropathy [12], vascular dysfunction [13], and cardiomyopathy [14]. However, the use of antioxidants such as vitamin C or E in clinical studies failed to show beneficial effects in diabetes [15,16], and research has even shown that excess supplementation with certain antioxidants might be harmful [17]. Therefore, research into the direct use of antioxidants or antioxidative herbal medicines should be carefully investigated.

Ginseng has been used as herbal medicine and a dietary supplement; it has various pharmacological beneficial properties [18,19,20]. Ginseng has also been used on patients with diabetes as an antidiabetic agent [21]. Ginsenoside Rd is a bioactive ingredient in ginseng and has renal preventive activities in cisplatin-induced renal dysfunction or ischemia-reperfusion renal injury rodent models [22,23,24]. Pectin-lyase-modified ginseng (GS-E3D) with enhanced ginsenoside Rd content has been newly developed [25,26]. This product has potent pharmacological activities on the glycation process [27], retinopathy [25], and nephropathy [7] in type 1 diabetic animal models. When the effects of GS-E3D were compared with those of an unmodified red ginseng extract on the glycation process and retinopathy, GS-E3D had a more potent inhibitory effect than that of an unmodified red ginseng extract [25,27]. However, this product has unknown effects on renal dysfunction in type-2 diabetic animal models. To elucidate this, we explored the renal preventative activity of GS-E3D in the db/db mouse model of type 2 diabetes. We also investigated the mode of action of GS-E3D both in vitro and in vivo. To the best of our knowledge, our work is the first to show that the antioxidative and antiapoptotic activities of GS-E3D result in the improvement of mesangial cell injury in vitro and renal dysfunction in vivo under diabetic conditions.

## 2. Results

### 2.1. GE-E3D and Ginsenoside Rd Alleviates ROS Generation in High-Glucose-Exposed MMCs

**Figure 1:**
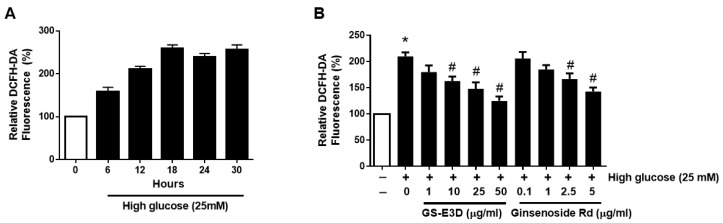
Effect of pectin-lyase-modified ginseng (GS-E3D) and ginsenoside Rd on oxidative stress in mouse mesangial cells (MMCs) under high glucose (25 mM). (**A**) The changes in oxidative stress levels under high glucose conditions were measured at each time point for 24 hours using DCFH-DA dye in MMCs. The fluorescence values are expressed as relative fluorescence levels compared with the control group. (**B**) The oxidative stress levels after the dose-dependent treatments of GS-E3D and ginsenoside Rd for 24 hours on MMCs under high glucose conditions. Data in figures are presented as the mean ± standard deviation (n = 4). * *p* < 0.05 vs. Control group; # *p* < 0.05 vs. High glucose-treated.

**Figure 2:**
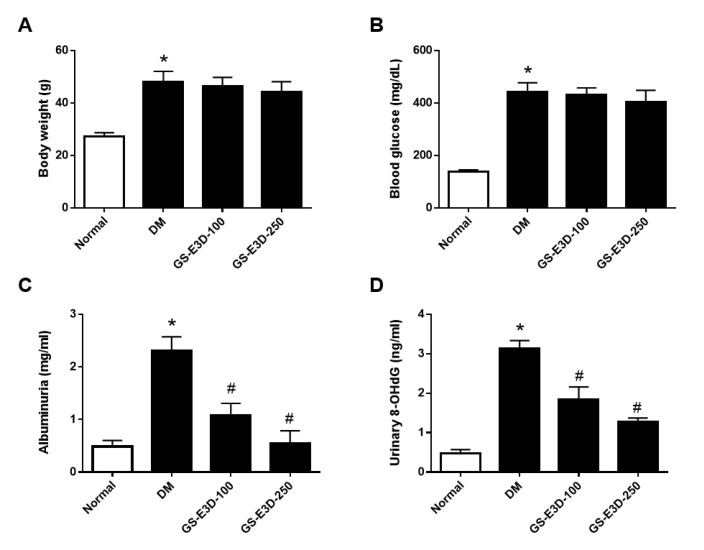
Effect of GS-E3D on physiological parameters in type 2 diabetic db/db mice. (**A**) Body weight. (**B**) Blood glucose levels. (**C**) Albuminuria. (**D**) Urinary 8-OhdG levels. Data are presented as the mean ± standard deviation (n = 8). * *p* < 0.05 vs. Normal group; # *p* < 0.05 vs. DM (diabetes) group.

Mouse mesangial cells (MMCs) were exposed to media containing 25mM glucose to find out the mechanisms of renal injury under high glucose conditions. First, oxidative stress in high-glucose-exposed MMCs was monitored for 24 h using 2’7’-dichlorofluorescin diacetate (DCFH-DA) (Figure 1A). DCFH-DA is commonly used to detect hydroxyl, peroxyl, and other reactive oxygen species. A fluorescence reading of DCFH-DA was largely increased by high glucose and peaked at 18 h. Cell survival rate was not significantly affected by GS-E3D treatment. MMCs were cultured in high glucose and various concentrations of GS-E3D or ginsenoside Rd were used as treatments for 24 h. DCFH-DA dye showed dramatically increased levels from high glucose, whereas the levels were significantly decreased by GS-E3D and ginsenoside Rd (Figure 2B). These results demonstrated that GS-E3D and ginsenoside Rd have an effect of decreasing oxidative stress in MMCs. It is well known that there are various ginsenosides in ginseng extracts. However, GS-E3D is a commercial pectin-lyase-modified red ginseng extract with an enhanced level of ginsenoside Rd. The level of ginsenoside Rd in GS-E3D was enriched 3-fold compared to an unmodified red ginseng extract [25]. Indeed, GS-E3D has shown more potent pharmacological activities in several animal experiments [7,25,27,28,29].

### 2.2. GS-E3D Improves Renal Dysfunction in Db/Db Mice

Diabetic db/db mice received two different doses of GS-E3D (100 and 250 mg/kg/day) daily for 6 weeks. Body weight (BW) increased in db/db mice and was not significantly changed by both doses of GS-E3D (Figure 2A). Blood glucose levels were not changed by GS-E3D (Figure 2B) but increased albuminuria was significantly recovered following GS-E3D administration (Figure 2C). Similarly, the urinary levels of 8-OhdG, an oxidative stress marker, were increased in the diabetes (DM) group and reduced by GS-E3D (Figure 2D). These data suggest that individuals with diabetes suffer renal dysfunction owing to increased oxidative stress, which can be alleviated by GS-E3D treatment.

### 2.3. GS-E3D Decreased ROS Production in Renal Tissues in Db/Db Mice

**Figure 3:**
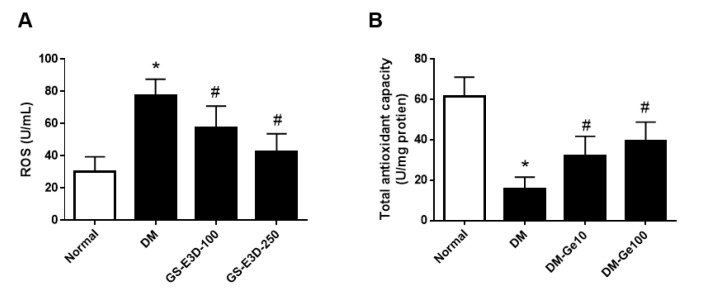
The levels of reactive oxygen species (ROS) (**A**) and total antioxidant capacity (**B**) in renal tissues were assessed by ELISA. Data are presented as the mean ± standard deviation (n = 8). * *p* < 0.05 vs. Normal group; # *p* < 0.05 vs. DM group.

Renal oxidative stress and antioxidant capacity levels were monitored under diabetic conditions (Figure 3). ROS production was largely increased, and antioxidant capacity was decreased in the db/db mice. GS-E3D treatment significantly prevented ROS overproduction as well as decreased the antioxidant capacity in db/db mice when compared with vehicle-treated db/db mice, to a level similar to that observed in normoglycemic lean mice.

### 2.4. GS-E3D Prevents Histopathological Changes and Apoptosis in Renal Tissues

**Figure 4:**
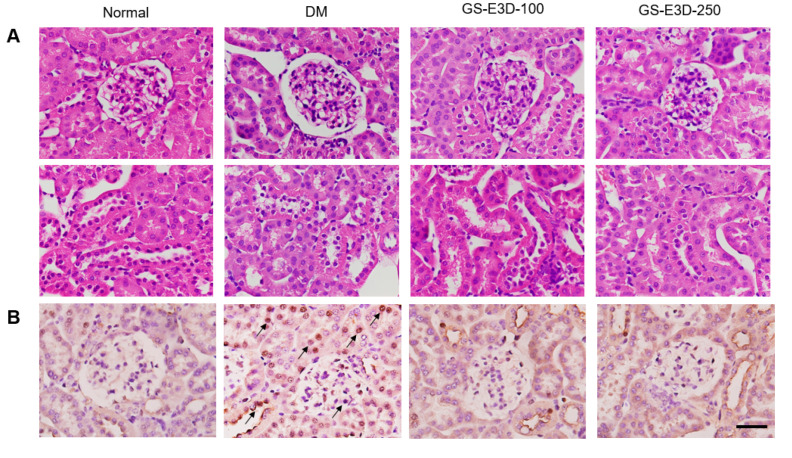

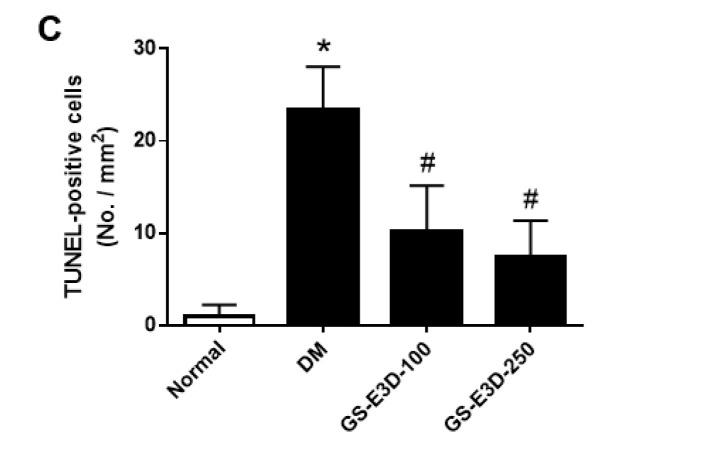
Effects of GS-E3D on histopathological alteration and apoptosis in renal tissues of db/db mice. (**A**) Histological analysis of renal tissues and images of hematoxylin and eosin (HE) staining. (**B**) TUNEL staining. Arrows indicate TUNEL-positive cells. (**C**) TUNEL+ cells counted from fluorescence microscopic images. Scale bar equals 50 μm. Data are presented as the mean ± standard deviation (n = 8). * *p* < 0.05 vs. Normal group; # *p* < 0.05 vs. DM group.

Hematoxylin and eosin (HE) staining revealed that the renal tissue from db/db mice showed histopathologic alterations such as glomerular mesangial expansion (Figure 4A, upper panels) and tubulointerstitial injury (Figure 4A, lower panels). However, when the db/db mice were treated with GS-E3D, these diabetes-induced histopathologic changes were significantly reduced. Apoptotic cells were detected by TUNEL (terminal deoxynucleotidyl transferase dUTP nick end labeling) staining and were increased in db/db mice and restored by GS-E3D (Figure 4B,C). Therefore, GS-E3D protects renal tissues and function in diabetes, not only by reducing oxidative stress but by inhibiting apoptosis.

### 2.5. Protective Effects of GS-E3D on Renal Tissues in Diabetes Were Dependent on Inhibition of Apoptosis-Related Protein Expression

**Figure 5:**
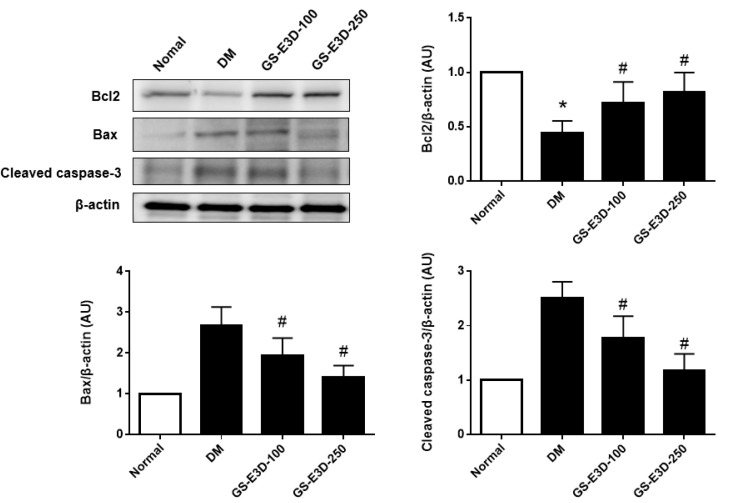
The expressions of apoptosis-related proteins. Relative protein levels were assessed by Western blot analysis. The blots of each protein were normalized to β-actin. All data are presented as the mean ± standard deviation (n = 6). * *p* < 0.05 vs. Normal group; # *p* < 0.05 vs. DM group.

In order to explore the protective mechanisms of GS-E3D in diabetes-induced renal injury, the protein expressions of apoptosis-related factors were analyzed in the renal tissues. Western blot analysis was performed by comparing each group to the DM group (Figure 5). Based on the collected data, the expressions of apoptosis-related proteins, including Bcl2, Bax, and cleaved caspase-3, are presented graphically, demonstrating that apoptosis was increased in diabetes and remarkably decreased following GS-E3D administration.

## 3. Discussion

Diabetes is an important risk factor associated with severe diseases through increasing blood glucose, oxidative stress, and chronic inflammation [30]. As type 2 DM, which accounts for most patients with diabetes, is caused by an imbalanced diet and lack of exercise, these patients are already exposed to various risk factors. Renal tissues are one of the tissues that are easily injured by high glucose conditions; previous studies have suggested that urine is a useful noninvasive indicator to assess the degree of oxidative stress in the pathophysiologic status of diabetes [31]. Clinically, the dysfunction of kidneys is frequently found in diabetes, and these people have a higher incidence of chronic renal failure [32]. Therefore, the dysfunction of kidneys in diabetes should be managed in order to regulate blood glucose and maintain quality of life. In this study, we assessed GS-E3D as a dietary supplement for the dysfunction of kidneys in diabetes through antioxidative and antiapoptotic activities, regardless of its blood-glucose-lowering function.

Ginseng has a beneficial activity on hyperglycemia [21]. However, GS-E3D failed to improve hyperglycemia in db/db mice. Previously, red ginseng was shown to lower blood glucose levels in a diabetic rodent model [33], with an oral dose of 1025 mg/kg/day. In the present study, the highest dose of GS-E3D was 250 mg/kg/day, about 5-fold less than that of the previous study. Interestingly, our results suggest that GS-E3D has a renal-dysfunction-preventive effect that is independent of glycemic control.

MMCs show an increase in oxidative stress under high glucose conditions. These results demonstrate that glomerular mesangial cells are vulnerable to oxidative stress induced by high glucose. Ginseng and its bioactive ingredient, ginsenoside Rd, have shown antioxidative effects in various cells [34,35]. Therefore, the protective effects of GS-E3D were expected in hypofunctioning kidneys induced by diabetes. It was previously reported that red ginseng had antioxidant activity [34,35], and GS-E3D also reduced the urinary excretion of 8-OhdG, an oxidative DNA damage marker, in streptozotocin-induced type-1 diabetic rats [7]. In our in-vitro and in-vivo experiments, renal oxidative stress induced by high glucose was dose-dependently reduced by GS-E3D. Oxidative stress plays a harmful role in diabetes-induced renal injury, and its inhibition had beneficial effects on the progression of diabetic nephropathy [36]. The dysfunction of mouse mesangial cells induced by high glucose was improved by antioxidants [37]. Consistent with these previous reports, our results showed that the antioxidative activity of GS-E3D resulted in the improvement of mesangial cell injury in vitro and renal dysfunction in vivo under diabetic conditions.

In the present study, db/db mice were selected because of the many similarities to human type-2 diabetes. Moreover, the development of diabetic nephropathy in db/db mice is accompanied by functional and morphological kidney damage that resembles human diabetic nephropathy [38]. The db/db mice showed mesangial matrix expansion and tubulointerstitial damage. These histological changes are considered a hallmark of diabetic nephropathy [39]. In the present study, we have further revealed that many podocytes, mesangial cells, and tubular cells are positively labeled by the TUNEL staining. The increase of apoptotic cells in db/db mice is associated with increased albuminuria. The apoptotic cell loss of glomerular cells has been demonstrated to correlate with worsening albuminuria [40]. Furthermore, strong evidence has established a role for intracellular ROS as potent inducers of glomerular cell apoptosis [41].

In this study, GS-E3D also inhibited the enhanced expression of apoptosis-related proteins, such as Bax, Bcl-2, and caspase-3, in db/db mice. Bax and Bcl-2 have an important role in determining cell survival versus cell death [42]. The altered expressions of Bax and BCL-2 under diabetic conditions resulted in apoptosis [43]. This alteration of the Bax/Bcl-2 ratio induced a release of cytochrome *c* from mitochondria, leading to the activation of caspase-3 [44].

The mode-of-action of ginseng on diabetes-induced renal dysfunction is still unclear. In previous studies, red ginseng inhibited the formation of glycation products in the renal tissues of diabetic animals [45]. Ginsenoside Rd had proliferation inhibitory activity on mesangial cells [46]. Although the effects of GS-E3D on diabetes-induced renal dysfunction were not compared with those of an unmodified ginseng extract, our previous report showed that GS-E3D had more potent antiglycation activity than that of an unmodified ginseng extract. GS-E3D, with an enhanced concentration of ginsenoside Rd, may have more potent renal-dysfunction-preventive activity.

GS-E3D was extracted with hot water, which is the method used traditionally. The GS-E3D used in this study consists of 60% dried red ginseng extract and 39.5% water [47]. The daily dose of GS-E3D (250 mg/kg/day) for a 60kg person is about 1250 mg/day, approximately 2 g of raw ginseng root per person. GS-E3D has an excellent safety record [47]. Therefore, we suggest that the clinical use of GS-E3D could be safe and not toxic to human health.

Based on all these results, GS-E3D has protective effects for diabetes-induced renal dysfunction by reducing oxidative stress and inhibiting apoptosis in renal tissues. These results provide a basis to choose red ginseng as a dietary supplement for patients with diabetes experiencing renal dysfunction.

## 4. Materials and Methods

### 4.1. Preparation of Pectin-Lyase-Modified Ginseng

Dried *Panax ginseng* C. A. Mey. was obtained from Wooshin Industrial Co. Ltd. (Geumsan, Korea). Red ginseng extract was incubated with 10% pectin lyase (Novozyme, Denmark) at 50 °C for 5 days. The extract was then heated at 95 °C for 10 min, freeze-dried, and stored at 4 °C until use. The contents of ginsenosides in the extract were determined by HPLC analysis. HPLC analysis was performed with an Agilent 1200 HPLC instrument (Agilent Technologies, Santa Clara, CA, USA). The pectin-lyase-modified ginseng extract contained 1.55% ginsenoside Rb1, 0.95% ginsenoside Rb2, 0.99% ginsenoside Rc, 1.56% ginsenoside Rd, 0.64% ginsenoside Re, 0.22% ginsenoside Rf, and 0.33% ginsenoside Rg1.

### 4.2. Cell Culture

Mouse kidney mesangial cells (MMC) was obtained from American Type Culture Collection (Manassas, VA, USA) and cultured in Dulbecco’s modified Eagle’s medium (Gibco, Carlbad, CA, USA):F-12 (3:1) containing 5% fetal bovine serum and 14 mM HEPES. For high-glucose conditions, the culture medium was exchanged with a serum-free medium containing 25 mM of glucose. Cell viability was examined manually by MTT assay. Briefly, cells were plated (1 × 10^4^ cells/well) in triplicate into 96-well plates for 24 h, and the mediums were exchanged with different concentrations of GS-E3D or ginsenoside Rd and incubated for 24 h. Then, 10 μL of 5 mg·mL^−1^ of MTT solution was added into each well and the plate was incubated at 37 °C for 3 h. The medium was discarded and DMSO was added to dissolve the MTT formazan; absorbances were then detected at 540 nm. The values are presented as a relative change compared to control cells.

### 4.3. Oxidative Stress Assay in MMCs

Oxidative stress was detected using DCFH-DA staining (Invitrogen, Carlsbad, CA, USA) according to the manufacturer’s instructions. MMCs were plated (1 × 10^4^ cells/well) in triplicate into 96-well plates for 24 h, and the medium was exchanged with serum-free medium, with 25 mM glucose mixed with different concentrations of GS-E3D or ginsenoside Rd for 24 h. The levels of oxidative stress are presented as a relative change compared to control cells.

### 4.4. Animals

Seven-week-old male type-2 diabetic db/db and nondiabetic db/+ lean mice were purchased from Japan SLC (Shizuoka, Japan). Including the normal group, a total of 4 groups were examined: (1) normal mice (Normal, n = 8); (2) diabetic (DM, n = 8) mice; (3) DM mice treated with GS-E3D 100 mg/kg body weight (GS-E3D-100, n = 8); (4) DM mice treated with GS-E3D 250 mg/kg body weight (GS-E3D-250, n = 8). GS-E3D was orally administered daily for 6 weeks. The animal experiment was conducted according to a procedure approved by our Institutional Animal Care and Use Committee (IACUC, Approval No.: 15–100). Blood was collected from the tail vein, and hyperglycemia was determined using a glucometer (Accu-Chek, Roche Diagnostics, Berlin, Germany).

### 4.5. Bioassay of Urine

To collect urine samples, all rats were kept in metabolic cages for 24 h. The urinary concentrations of albumin and 8-hydroxy-2′-deoxyguanosine (8-OhdG) were determined using a mouse albumin ELISA kit (Abcam, Cambridge, UK) and an 8-OHdG Check ELISA kit (Cosmo Bio, Tokyo, Japan), respectively.

### 4.6. Oxidative Stress Assay on Kidneys

In brief, renal tissues were homogenized, and the levels of reactive oxygen species (ROS) and total antioxidant capacity were assayed using a mouse reactive oxygen species ELISA kit (MyBioSource, San Diego, CA, USA) and a total antioxidant capacity assay kit (MyBioSource, CA, USA), respectively, according to the manufacturer’s instructions.

### 4.7. Hematoxylin and Eosin Staining

Formalin-fixed renal tissues were embedded in paraffin and sliced in 4-μm thick sections. The sections were deparaffinized and rehydrated using standard techniques and stained with Mayer’s hematoxylin and 1% eosin. The stained sections were dehydrated and cleared with xylene. Mounting was done by using canada balsam and observed in a light microscope.

### 4.8. TUNEL Staining

The apoptotic cells on the tissue were detected by terminal deoxynucleotidyl transferase (TdT)-mediated dUTP nick end labeling (TUNEL) staining that was performed according to the manufacturer’s instructions (Promega, Madison, WI, USA). Briefly, tissue slides were pretreated with 20 μg·mL^−1^ proteinase K solution and incubated with the mixture containing TdT and fluorescein-12-dUTP for 1 h at 37 °C. Nuclei were counterstained by a mounting medium containing DAPI. Apoptotic cells were observed in a fluorescence microscope.

### 4.9. Western Blot Assay

The renal tissues were homogenized using RIPA lysis buffer. The protein concentrations were determined by Bradford assay (Bio-Rad, Hercules, CA, USA) according to the manufacturer’s instructions. The protein samples were loaded onto 12% polyacrylamide gels and transferred to PVDF membranes after electrophoresis. The primary antibodies used here were anti-Bax, anti-Bcl2, anti-cleaved caspase-3, and anti-β-actin antibodies (Santa Cruz Biotechnology, Santa Cruz, CA, USA). The immune complexes were then visualized with an enhanced chemiluminescence detection system (WesternBright™ ECL, Advansta, CA, USA). Protein expression levels were determined by analyzing the signals captured from the PVDF membranes with an image analyzer (Lumino GraphⅡ, ATTO Corporation, Tokyo, Japan).

### 4.10. Statistical Analysis

The data in all figures are presented as the mean ± standard deviation. Significant differences between groups were determined using one-way ANOVA, followed by Tukey’s posthoc test. Differences were considered statistically significant at *p* < 0.05.

## 5. Conclusions

Our study shows that GS-E3D has protective effects on diabetes-induced renal dysfunction by reducing oxidative stress and inhibiting apoptosis in renal tissues. The findings herein suggest that GS-E3D may help patients with diabetes experiencing renal dysfunction when taken as a dietary supplement. To obtain approval of GS-E3D as a functional food or dietary supplement in Korea, further research is needed to clarify the clinically beneficial effects of GS-E3D on human subjects. We will conduct a human clinical trial based on the results of this in-vivo study.

## Data Availability

The data will be available on request.

## Sample Availability

Samples of the compounds at amounts less than 1 mg are available from the authors.
